# A Retrospective Survey of Factors Affecting the Risk of Incidents and Equine Injury During Non-Commercial Transportation by Road in the United Kingdom

**DOI:** 10.3390/ani10020288

**Published:** 2020-02-12

**Authors:** Carol Hall, Rachel Kay, Jim Green

**Affiliations:** 1 School of Animal, Rural and Environmental Sciences, Nottingham Trent University, Brackenhurst Campus, Southwell, Nottinghamshire NG25 0QF, UK; 2 British Animal Rescue and Trauma Care Association CIC, The Horse Trust, Slad Lane, Speen, Princes Risborough, Buckinghamshire HP27 0PP, UK; jim.green@bartacic.org

**Keywords:** horse, equine, transport, transportation, horsebox (lorry truck), trailer (float), survey, accident, injury

## Abstract

**Simple Summary:**

The transport of horses by road is necessary for several reasons, including competition and leisure, moving horses between yards and for breeding and veterinary purposes. In addition to the risks associated with road travel in general, the reaction of some horses to confinement in a transport vehicle may result in injury to the animal. An online survey was carried out to investigate the frequency of incidents during road transport and identify potential risk factors. Of the 2116 survey participants, 342 reported incident details. Over 50% of these incidents were attributed to the behaviour of the horse during transport, with most of these occurring during the first hour of the outward journey. The horse was injured in over 50% of the incidents, with transport vehicle malfunction being thought to be responsible for 68% of these injuries. Those transporting horses for competitive or professional purposes were more likely to have reported an incident than those transporting for leisure purposes. The findings of this survey highlight the need for better training and preparation of horses for transportation and to identify the risk factors associated with transport vehicles.

**Abstract:**

The number of equines injured as a result of incidents during road transport is currently unknown in the United Kingdom. Although previous research has identified factors that affect an equine’s behavioural and physiological responses to transportation, their contribution to incident occurrence and injury risk is unclear. The aim of this study was to identify factors associated with incident occurrence and equine injury during transportation by road. An online survey was administered between 12 May 2017 and 21 July 2017 in the UK. The survey was open to those transporting equines non-commercially and comprised two sections. Questions relating to general transport behaviour were completed by all participants. Participants who had experienced an incident then provided details of these, including outcomes. Incidents were reported by 16.2% (342/2116) of participants, with details included for 399 incidents. Those participants who had a professional/competitive involvement with equines reported more incidents than those with a predominantly leisure involvement (*p* < 0.01). Equine behaviour was the attributed cause of 56% of incidents reported and most incidents occurred during the first hour of travel (65%). In over 50% of the incidents reported, the equine was injured, with those incidents attributed to transport vehicle malfunction being associated with the highest percentage of injury (68%). This study highlights the need for better preparation of the equine for transportation and to identify risk factors associated with transport vehicle type, design and operation.

## 1. Introduction

The transport of horses by road is necessary for several reasons, including competition and leisure, commercial activity, and for breeding and veterinary purposes [1]. The conditions associated with the commercial transport of horses for slaughter have been shown to compromise welfare and frequently result in equine injury, causing both physiological and psychological distress [2,3,4]. Although the conditions under which horses and other equines are transported for sporting and leisure purposes are not directly comparable, research findings to date suggest that such transportation may still have a negative impact on health and performance [5]. The scale of the problem and the prevalence of injuries sustained during non-commercial transportation for sport, leisure and related purposes has yet to be determined. Recent surveys conducted in Australia [6] and New Zealand [7] indicate that transport-related incidents in the equine sporting and leisure sectors are not uncommon, with the potential scale of the problem dependent upon transportation frequency [7]. In the United Kingdom (UK), an online survey of horse owners found that approximately 60% of respondents regularly transported their horse to attend events and activities [8] and a cross-sectional UK study found that, out of a sample of 797 survey respondents, 22.5% had transported their animals within the previous week (54.7% in a trailer, 41.3% in a horsebox, and 3.9% used both) [1]. A survey conducted by the British Equestrian Trade Association (BETA) in 2015 estimated that there were 1.3 million riders in the UK, approximately 944,000 horses and 446,000 horse-owning households [9]. A conservative estimate based on these survey findings [1,8,9] suggests that 25,000 animals are transported regularly for non-commercial purposes by road in the UK. 

A high prevalence of traumatic injury has been found to occur in horses in general [5]. In a UK survey, 40% of horse owners reported that their horse had sustained at least one injury within the previous twelve months [10]. The risk of injury was associated with the type of horse (cobs and ponies were less likely to have sustained an injury than other types), use (horses used for competition were more likely to have sustained an injury) and age (older horses were less likely to have sustained an injury) [10]. Some horses left alone in a field were reported to have exhibited behavioural signs of distress that resulted in injury [10]. Although, in this study, only 2% of the injuries sustained were associated with transportation, compared with 62% in the field and 13% when ridden [10], the risk of transport-related injury should not be underestimated. In Australia, a survey of horse injury during non-commercial transport that was conducted at competitive events found that 24.7% of participants reported transport-related injuries to their horses [6]. In New Zealand, in a recent study of the human factors associated with equine road transport issues, 17.7% of survey participants reported that they had experienced at least one transport-related horse injury over the past two years [7]. Those with a professional involvement with horses were found to have experienced more incidents than those with an amateur involvement, with the increased frequency of travel as well as the greater number of horses managed by those in the professional sector being suggested as contributory factors [7]. 

Transport-related factors found to be associated with the risk of injury include the number and type of horses being transported within the vehicle, the type of vehicle, internal vehicle fittings and vehicle maintenance and, in long-haul transportation, the length of the journey. Horses transported commercially in groups are at risk of injuries sustained from the aggressive behaviour of individual animals, as well as falls and balance issues worsened by a lack of space. In horses transported by road for slaughter in Canada, injuries associated with inter-horse aggression (kicks and bite-related injuries) and trauma were found [4]. Of the 100 horses examined, 33% had injuries that were visible, 48% had areas with a raised surface temperature (identified by thermography) and 72% had bruising (identified by carcass examination) [4]. Increased journey length was found to be associated with an increase in the occurrence of visible injuries, although increased density was not [4]. However, increased stocking density during the transport of groups of feral ponies was found to be associated with increased aggression, as well as balance issues, collisions and falls [11]. The ability of fallen animals to get up may be limited by lack of space and other animals, resulting in an increased risk of injury [12]. Although most animals transported non-commercially for recreation and sporting purposes are not transported in groups, the horse-carrying capacity of the vehicle was found to show some association with an increased risk of injury in the Australian survey conducted by Riley et al. [6]. The impact of inter-horse aggressive behaviour is likely to be lessened during non-commercial transport where most animals are segregated, but this will vary according to the internal design of the vehicle.

Several transport-related factors have been identified as potential stressors that may contribute to compromised equine welfare, behavioural problems and injury during transport [13,14,15]. Physical stressors include the motion of the vehicle and features of the flooring, ambient temperature and humidity, and restricted space [16,17,18]. The ability of the horse to maintain its balance during vehicle movement was found to be affected by traffic and road conditions, the condition of the transport vehicle, and by driving style and experience [16]. In the survey conducted in Australia, most transport-related injuries (83.6%) were found to occur while the vehicle was moving, with over 50% involving the lower limbs, indicative of balance issues [6]. The ability to maintain balance has also been found to vary according to the orientation of the horse, although conclusions regarding the optimum direction of travel vary. Some findings indicate a preference for rear-facing travel [19,20], others for a 45° orientation [21]. Individual variation in preferred orientation has also been found [22]. Factors shown to cause anxiety during transportation, such as isolation [23], and adaptation to the transport environment during the first hour of the journey [14,24] may also relate to an increased risk of incidents and equine injury. Further research is required to determine whether there are other factors associated with the risk of transport-related injuries and the global scale of the problem. Within the European Union (EU) there is legislation to protect the welfare of animals during transport (regulation (EC) No. 1/2005), and in the UK *‘it is an offence to transport any animal in a way which causes, or is likely to cause, injury or unnecessary suffering to that animal’* (Welfare of Animals (Transport) (England) Order 2006) [25]. In terms of both compliance with legislation and the protection of equine welfare, further consideration of equine transport practices is required. 

Although several factors have been shown to impact on equine stress-related behaviour and compromised welfare during road transport, their relationship to the occurrence of incidents and resultant equine injury is unclear. However, some aspects of equine behaviour during transportation undoubtedly increase the risk of injury to both horse and human [26]. A survey investigating transport-related problem behaviours found that habituation to the transport situation reduced the risk of equine injury during transportation [27]. There is currently more focus on training in preparation for loading horses into transport vehicles than on preparing for the actual journey [27,28], but the results of a survey of horses exhibiting trailer problems indicated that problems associated with travelling were only slightly less prevalent (51.5%) than problems associated with loading (53.4%) [29]. 

Replication of the non-commercial transport experience in an experimental situation is challenging and would not reflect the variety of road conditions experienced in reality [5]. Consequently, a retrospective survey-based approach has been used in previous studies to evaluate the risk of injury during equine transport [6,7,27,29,30]. To date, surveys aimed at evaluating these risk factors in the non-commercial transport of equines have been conducted in Australia [6,27,30], New Zealand [7] and the United States [29]. In the UK, horses are transported in two types of vehicle (trailers that are towed or motorised horseboxes) which may be associated with specific risk factors. The aim of the current study was to retrospectively identify factors that had been associated with incident occurrence and injury in equines transported non-commercially within the UK, including the risks associated with the two different forms of transport. 

## 2. Materials and Methods 

The study was approved by the Nottingham Trent University’s Joint Inter-College Ethical Committee.

### 2.1. Respondents

The target population for this survey were owners, riders and trainers in the UK with experience of transporting equines, either by themselves or by a third party. Respondents were required to be aged 18 years or over, anonymity was assured, and the subsequent analyses did not include a reference to any individual or organisation. Individual respondents were recruited via social media, e-mail and equestrian societies, including the British Horse Society. Based on an estimated target population of 25,000 UK equine industry participants [8,9], ≥1023 surveys were required to attain a 95% confidence level and an error level of ±3% [31]. 

### 2.2. Survey

The survey content and design were informed by the findings of a preliminary survey into the factors associated with incidents during the road transport of equines conducted by the British Animal Rescue and Trauma Care Association (BARTA) [32]. A pilot survey was completed by volunteers with experience of equine transport (n = 3) to inform the guidance provided for respondents and ensure that the design enabled respondents to complete the survey. The survey was administered between 12th May 2017 and 21st July 2017 using the Bristol Online Survey tool. (Supplementary Material S1: survey questionnaire). Respondents were informed that the survey included questions about their experience of transporting equines and about measures that could be taken to reduce the associated risks. Equines included horses, ponies, donkeys and mules (referred to within the actual survey as ‘horses’, as noted within the respondent information). 

Respondents were also informed that it would take approximately 20 min to complete the first section plus 20 min per incident reported. Respondents were requested to have the following information available before starting the survey:

- Driver transport qualifications (if any);
- Vehicle (lorry and/or trailer) information;
- Details of any transport incidents/accidents.

They were asked to select ‘don’t know’ for questions for which they did not have full details. 

The survey included two main sections: 

*Section 1.* The first section included questions relating to general transport behavior, which were used to compile a demographic profile of who transports equines by road, how and why. All respondents were also asked whether they had experienced an incident or near miss during transport. This section comprised questions relating to the details of the respondent, the driver and vehicle, and typical journey details. Responses to the questions relating to knowledge of commercial transport and its use (n = 5) were not included in the current analyses. The demographic variables and response categories used in the subsequent analyses are shown in Table 1. The respondents were asked whether they had experienced an incident or near miss while transporting equines. The term incident referred to events that were construed as accidents that had occurred during transportation; for example, horse injured itself inside the vehicle or a collision with another road user. The term near miss referred to events that could have resulted in an incident or accident occurring but did not. The type of near miss was categorised by the respondent as driver error, other road users, equine behaviour, vehicle malfunction or other factors. No further details of these near misses were requested. Those respondents who had reported having experienced an incident were then directed to *Section 2* of the survey to provide details of the incident.

*Section 2*. The second section of the survey asked for details of specific incidents (details provided were specific to that incident and separate from the demographic details). Each respondent could add details for up to five separate incidents. Driver and vehicle details, time and type of incident, and the outcome were requested. Only those respondents who had reported experiencing an incident were directed to *Section 2*. This section comprised questions relating to details of specific incidents and their outcomes. No time frame was specified for the incidents reported, but respondents were asked to provide the (approximate) date on which the incident occurred. This open-ended time frame was included to enable data to be collected relating to the long-term consequences of reported incidents in relation to horse performance and equine welfare. The incident variables and outcomes with response categories are shown in Table 2.

### 2.3. Data Collection and Analyses

The survey responses were exported from the Bristol Online Survey as coded Excel files. Where responses resulted in categories with < 5% of the population values, related categories were combined to enable statistical analyses. Within the demographic data, the categories breeder, competitor, racing/point-to-point and professional were combined (P), as were leisure and parent of child who rides (R). Within the incident data, the following reasons for travel were combined as competition/training: racing, local, national and international shows, training. Veterinary, breeding and moving horses between yards were combined as maintenance. Within the category ‘Type of equine being transported’, horse and donkey was combined with horse and pony (see Table 1 and Table 2). All statistical analyses were conducted using IBM SPSS Statistics (25) software. Significance levels of *p* ≤ 0.05 were used in all statistical analyses.

Descriptive statistics of the categorical data (including reported near misses) were reported as the frequency and percentage of responses (and missing values where these occurred). The data from Section 1 of the survey (completed by all respondents) and Section 2 of the survey (completed only by respondents who reported having experienced an incident during equine transportation) are presented separately.

From the survey population demographic data (Section 1), the association between demographic characteristics (explanatory variables) and reported incidents (outcome) and near misses were investigated (separately). Univariate logistic regression analyses were conducted to identify significant associations between the explanatory variables and the outcome (incident reported yes/no; near miss reported yes/no). Odds ratios (OR), including 95% confidence intervals (CI), were calculated for each category of the variables found to be significantly associated with the outcome. The associations between the significant variables were tested (Pearson’s Chi-square test) prior to the inclusion of significant variables in the subsequent multivariate logistic regression analysis (using the forced entry method, where all predictor variables were tested in one block to assess their predictive ability while controlling for the effect of other predictors in the model). Where categories had expected counts of <5, these were either amalgamated or removed from the analyses.

From the incident data (Section 2), descriptive statistics were reported as the frequency and percentage of responses relating to incident characteristics and outcomes. Where the respondent had answered ‘don’t know’, this was regarded as missing data for subsequent analyses. Significant variation in the frequency of categories within incident characteristic variables, when compared with those in the survey population, were explored using a one sample Chi-square test. Univariate logistic regression analyses were conducted to identify significant associations between incident characteristics and the outcome (equine injury yes/no). Odds ratios (OR), including 95% confidence intervals (CI), were calculated for each category of the variables found to be significantly associated with the outcome. Associations between incident and injury characteristics were explored using Pearson’s Chi-square test. The association between the significant incident variables and injury outcome was tested using multivariate logistic regression analysis (forced entry method). 

## 3. Results

### 3.1. Section 1

Section 1 of the survey was completed by 2153 respondents. Those who reported not being involved in the transport of equines by road (n = 37) were removed from the data set, leaving a respondent sample of 2116 for analyses. This number exceeded the estimate of ≥1023 required to attain a 95% confidence level and error level of ±3%. Table 3 shows the counts and percentage breakdown of response categories within the survey variables in Section 1 of the survey. The mode age range of the survey respondents was 26–55 years, mode gender was female, and mode involvement in the equine industry was for both recreational and competitive purposes. Trailers were more frequently used than motorised horseboxes (1044/2116, 49.3%), journeys were short (mode < 1 h), frequent (mode weekly or more) and most involved one equine being transported alone (1166/2116, 55.1%).

A total of 342/2116 (16.2%) of respondents reported having experienced an incident during equine road transport. Most of these respondents (290/342, 84.8%) only provided details for one incident, but details for 399 incidents were provided in total. Near misses were reported by 571/2116 (27%) of respondents. The most frequent type of near miss reported (293/571, 51.31%) involved other road users. Overall, 767/2116 (36.2%) of respondents reported having a near miss or incident, with 146/2116 (6.9%) reporting both. The counts and percentage breakdown of reported incidents and near misses (including the frequency of different types of near miss) are shown in Table 4.

The only significant association between demographic variables and the reporting of a near miss was found in relation to the involvement the respondent had with equines. The results of the univariate logistic regression analysis are shown in Table 5. Those respondents with a professional/competitive or multiple type of involvement with equines were more likely to have reported a near miss than those with a primarily recreational involvement. No additional details of the outcomes of near misses were requested in the survey.

The results of the variables from the univariate logistic regression analyses that were found to be significantly associated with the reporting of an incident are shown in Table 6. Those respondents with a professional or competitive involvement with equines were most likely to have reported an incident. Where multiple drivers were involved, and multiple reasons for transport, as well as frequent journeys, the odds of an incident having been reported were increased. Transporting equines for leisure purposes or using a commercial transport driver reduced the odds of the respondent reporting an incident. See Table 6 for details of the association between these variables and incident reporting, frequency and percentage of incident reporting for each variable category, and the odds of each category response reporting an incident. A strong collinearity was found between the variable journey frequency and the other variables: involvement in the equine industry (ꭗ^2^ = 155.34, df = 6, *p* < 0.001), driver identity (ꭗ^2^ = 166.09, df = 6, *p* < 0.001) and reason for transport (ꭗ^2^ = 67.08, df = 6, *p* < 0.001). As an increased frequency of travel increases the odds of experiencing an incident, this variable was excluded from further analyses. 

The results of the multivariate logistic regression analysis (ꭗ^2^ = 31.02, df = 6, *p* < 0.001) are reported in Table 7. The odds of those involved in the equine industry for recreational and leisure purposes only reporting an incident were lower than those who had competitive or professional involvement or were involved for multiple purposes. The exclusive use of commercial transporters reduced the odds of reporting an incident. Those respondents who transported equines for multiple purposes were twice as likely to have reported an incident than those who transported them for leisure/recreational purposes only.

### 3.2. Section 2 

Details for 399 incidents were reported by 342 respondents. Table 8 shows the counts and percentage breakdown of response categories for incident-specific driver and vehicle details, journey details and incident characteristics for these 399 incidents. Trailers were the most frequent vehicle involved in the incident (257/399, 64.6%). When compared with the survey population, where 49% respondents used trailers and 35% motorised horseboxes, and taking account of this within the analysis, significantly more trailers than lorries were involved in the incidents reported (ꭗ^2^ = 6.73, df = 1, *p* = 0.009). Most vehicles were owned by the respondent reporting the incident (299/399, 74.9%). Within the transport vehicle the partition was most frequently partial, with a gap above the floor (242/399, 60.7%), the animal was tied at eye level or above (237/399, 59.4%), and CCTV was only fitted in 78 (19.5%) vehicles. Horses were the most frequent type of equine involved (285/399, 71.4%), and in 255/399 (71.4%) incidents the equine was being transported alone. The most frequent type of incident involved horse behaviour (222/399, 55.6%), occurred when the vehicle was moving (269/399, 67.4%) and happened during the first hour of transport (261/399, 65.4%). A total of 219/399 (54.9%) of the incidents occurred during journeys made for competition or training purposes. The incident was considered to have been avoidable in 231/399 (57.9%) of cases. This was significantly associated with the type of incident reported (ꭗ^2^ = 26.38, df = 6, *p* < 0.001), with 20/23 involving transport vehicle malfunction being considered avoidable. 

The behaviour of the equine immediately prior to the incident (standing still, fidgeting or unknown) varied significantly in relation to the different types of incident (ꭗ^2^ = 42.67, df = 6, *p* < 0.001). Immediately before 91.7% (33/36) of incidents involving a road traffic collision, the equine was standing still. In 51.6% (115/222) of incidents attributed to equine behavior, the animal was fidgeting before the incident occurred. The identification of this pre-incident behaviour was significantly associated with the presence of CCTV in the vehicle (ꭗ^2^ = 45.25, df = 4, *p* < 0.001) with no cases of unknown behaviour reported where CCTV was available. No association was found between the presence of CCTV and the attributed incident type. Significantly more horseboxes (40.8%) than trailers (7.8%) were fitted with CCTV at the time of the incident (ꭗ^2^ = 65.61, df = 2, *p* < 0.001).

Further details of the attributed causes of incidents and the frequency of reported details of the specific type of occurrence, including differences between horseboxes and trailers, are provided in (Supplementary Material Table S1). Regional locations of incident occurrence in England, Scotland and Wales for those incidents for which UK location details had been provided (n = 234) are also provided in the (Supplementary Material Table S2).

The counts and percentage breakdown of incident outcomes (equine injury and recovery) in relation to the 399 incidents reported by survey respondents (n = 342) are shown in Table 9. 

In over 50% (206/399) of the incidents reported, the outcome included the equine being injured. The most common area of injury was the hind legs or multiple areas. In most cases, the injuries sustained were considered minor, but in 35 cases the equine did not fully recover from the injury. In most cases recovery time was between one week and six months. A significant association between the severity of injury and the presence of an internal partition within the vehicle was found (ꭗ^2^ =6.32, df = 2, *p* = 0.042). Expected cell counts were too low (<5) for further statistical analyses of factors associated with recovery from injury. 

The results of the variables from the univariate logistic analyses that were found to be significantly associated with injury as a result of the transport incident are shown in Table 10. 

The odds of horses being injured as a result of an incident during road transport were twice as high as for ponies. Injury was six times more likely in an incident involving transport vehicle malfunction than in a road traffic collision, and four times more likely when the type of incident was classed as relating to horse behaviour. Injuries were twice as likely to have occurred during the first hour of travel compared with later in the journey.

The results of the multivariate logistic regression analysis (ꭗ^2^ = 25.46, df = 5, *p* < 0.001) are reported in Table 11. The odds of a horse being injured in an incident were higher than ponies. Incidents occurring during the first hour of transport were more likely to have resulted in injury. Incidents involving road transport vehicle malfunction were associated with the highest odds of equine injury occurring.

## 4. Discussion

The findings of the current study carried out in the UK agree with those of comparable surveys conducted in Australia [6] and New Zealand [7], that those who transport horses for competitive and/or professional purposes were more likely to have experienced a transport-related incident than those transporting for leisure and recreation. This is in part likely to be a consequence of the increased frequency of travel, as well as the greater number of horses managed by those in the professional sector [7]. In Australia, in a survey of horse injury during non-commercial transport, 24.7% of participants reported transport-related injuries to their horses [6]. This slightly higher percentage than that found in the current study could be linked to the fact that the survey was conducted at competitive events and, again, the participants were likely to transport their horses frequently. As this is unavoidable for those involved in professional and competitive equine pursuits, and transport is necessary for other purposes [1], it is important to identify factors where changes can be made to reduce the risk of incidents and related equine injuries occurring. In addition to the number of respondents reporting incidents in the current study (16.2%), a further 20.1% reported experiencing a near miss. The wording of this question was likely to have biased the types of near misses being reported (51.31% attributed to other road users, compared with 9.02% of reported incidents attributed to road traffic issues/other road users) but the overall frequency supports the conclusion that there are considerable risks associated with transporting horses by road for non-commercial purposes. Given the concurrence between the findings of studies carried out in different nations, there are underlying issues that need addressing internationally.

The impact of vehicle type on the likelihood of an incident occurring and the subsequent severity of the outcome for the horse has yet to be fully determined. In the current study, incidents were more likely to have involved trailers as opposed to motorised horseboxes, although the risk of subsequent injury did not vary with vehicle type. In an online survey carried out in New Zealand, transport-related behaviour problems were found to vary according to vehicle type, which also impacted on transport practices, including driver behaviour [33]. Trailers are more commonly used in the UK than motorised horseboxes, and there are legislative differences in the required maintenance for each type of vehicle, as well as differences in driver training and qualification requirements. It has been shown that driving style and vehicle condition both affect the ability of the horse to maintain its balance during transport [16]; such differences in relation to vehicle type may contribute to incident occurrence. For example, in the UK motorised horseboxes are subject to annual inspections, whereas trailers are not. In the current study, the service history of the trailers involved in 55 (13.8%) incidents was unknown and in five (1.3%) the trailer had never been serviced. Incidents involving transport vehicle malfunction, although least frequent (5.5% of incidents reported), were the type of incident most likely to result in equine injury. The need for a review of transport vehicle maintenance, at least in the UK, is indicated. An international review of animal transport maintenance requirements and driver training, together with an audit of related incidents and injury on a larger scale, would further inform the importance of this aspect of transportation and its impact on equine safety. Internal fittings within these vehicles should also be reviewed. For example, internal partitions were fitted in 94% (375) of the vehicles (trailers and horseboxes) involved in incidents in the current survey and were found to be associated with the severity of incident-related injuries. No distinction between trailers and horseboxes in relation to the type and/or impact of partitions on incident outcomes was found in the current study, but further assessment of internal vehicle design is warranted. 

An increased risk of equine injury during transport has been associated with transport-related behaviour problems [26]. Riley et al. reported that a high proportion (75%) of road transport incidents were associated with the behaviour of the horse, including scrambling, slipping and horse–horse interactions [6]. Equine behaviour was attributed as the cause of a slightly lower proportion of incidents in the current study (55.6%), but both sets of findings suggest that measures should be taken to reduce the adverse reactions of equines to travel. Behavioural issues are most likely to occur during the first hour of travel as the horse adapts to the transport environment and the motion experienced during travel [14,24]. In the current study, it was found that injuries were twice as likely to have been sustained in incidents occurring during the first hour of travel compared with later in the journey, suggesting that additional measures should be taken to help the horse adapt to the transport environment. Ensuring that the horse is habituated to the transport environment has been shown to reduce the risk of injury [27] and non-aversive training in preparation for the situation would reduce behavioural signs of anxiety [28]. An additional stressor during transport is isolation when transporting single animals [23]. In the current study, 63.91% of incidents involved equines being transported on their own. Preparation for this aspect of travel or, ideally, the provision of a companion (or surrogate companion, such as the use of a mirror) should be considered to reduce the negative impact of isolation during transport [23]. As found in the UK survey of factors affecting the occurrence of traumatic injuries sustained by equines in general [10], the current survey results showed horses to be twice as likely to sustain an injury during an incident than ponies. Further investigation is needed to determine whether this is a consequence of different behavioural tendencies, size in relation to vehicle dimensions or other factors. The use of CCTV to monitor equine behaviour during transport facilitated the recognition of unsettled behaviour but devising measures that should be taken to avert a subsequent incident is a major challenge, particularly when travelling on a motorway or somewhere where stopping is not an option. In addition to increasing the number of vehicles fitted with CCTV and ensuring that drivers and their assistants can recognise equine behavioural signs of unease, an effective means of calming such animals during transport would be invaluable. Although sedation is used in some cases, this can reduce the ability of the horse to maintain its balance [34] and is not an option when transporting for ridden work. Furthermore, CCTV footage requires monitoring by the assistant rather than the driver to prevent distraction that could result in driver error [35].

This study investigated whether factors relating to the way in which equines are transported by road non-commercially within the UK were associated with the likelihood of an incident occurring during this activity. Also, whether the risk of injury because of these incidents was associated with factors such as the type of equine involved, the type of vehicle and features of the journey during which the incident occurred. The number of survey responses obtained in the study ensured a statistically representative sample of those transporting equines non-commercially within the UK. However, the open-ended timescale used to facilitate the collection of data relating to the longer-term consequences of road transport incidents and injury meant that frequency estimates could not be reliably calculated from the data. Despite this limitation, the study findings, that in over 50% of the incidents reported the equine involved was injured, with 17% of the injured animals never fully recovering, highlight the importance of identifying and addressing associated risk factors. 

The data for this study were collected by means of a survey and the potential bias in respondent participation should be considered when interpreting the results [36]. The self-selection by participants is likely to have attracted those with negative experiences of transporting horses by road, although the percentage of respondents reporting incidents was comparable with other studies. The open-ended timescale for incidents to be reported made it possible that the more serious incidents were remembered and reported more frequently than less serious ones, and that details of incidents may not have been recalled accurately. The fact that the survey was administered online will have introduced a bias towards respondents familiar with, and with access to, the internet. Survey distribution was via social media and organisational promotion, so could not be considered random. Potentially the most important limiting factor in relation to this survey was its length. The details requested for each incident were extensive and it is likely that the time required to complete it will have reduced the number of incidents reported. However, enough responses were collected to ensure a representative sample and draw some initial conclusions relating to incident occurrence and injury during the transport of equines by road. 

The findings of this study provide initial insights into factors that are associated with the occurrence of incidents during the transport of equines by road in the UK. Although the data cannot be used to accurately estimate the number of incidents occurring, or identify causation, the results of the survey do provide an indication of the proportion of incidents that result in injury and factors that were associated with this outcome. Concurrence with the findings of similar studies conducted in other nations implies that there are generic issues to be addressed in order to comply with international animal transport legislation and to reduce the negative impact of transport on equine welfare. 

## Figures and Tables

**Table 1:** Name and description of demographic variables and response categories.

Name	Description	Categories
*Respondent details*		
Age		<26 yrs, 26–55 yrs, >55yrs
Gender		Male, female, unspecified
Involvement with equine industry	Responder’s main involvement with equines	Recreation (R), professional (including competitive) (P), multiple (M)
*Driver and vehicle details*		
Driver identity	The most frequent driver of the transport vehicle	Self, commercial, multiple drivers
Driver training	Training undertaken at any level, including practice with experienced driver and training for specific qualifications	Yes; no
Driver qualifications	Driver holds UK qualifications relating to the transport of animals (Includes B+E, CET, ACET, CPC, HGV, WATO, multiple)	Yes; no
Transport vehicle	Main type of vehicle used to transport equines	Trailer, motorised horsebox, both, commercial transport only
*Journey details*		
Reason for transport	The main reason the respondent transports equines	Leisure and recreation (including moving location and pony club activities), competition and training (including related professional activities), multiple
Number of animals transported	Most common number of animals transported together by the respondent	One, two, >two
Frequency of journey	Frequency with which equines are transported by the respondent	Weekly or more, every 2-4 weeks, less than once a month, varies
Duration of journey	Most common duration of journey undertaken by the respondent	<1 h, 1–4 h, >4 h

**Table 2:** Incident variables and outcomes including response categories.

Name	Description	Categories
*Driver and vehicle details*		
Vehicle		Trailer, motorised horsebox
Vehicle owner		Own, commercial, borrowed/rented, friend
Driver qualifications	Transport specific qualifications held by driver	Yes, no, don’t know
Horsebox size	Gross vehicle weight (horseboxes only)	>3.5 tonnes, ≤3.5 tonnes, don’t know
Trailer servicing	Time since trailer was last serviced (trailers only)	Within last 6 months, 7–12 months before, 1–5 years before, never serviced, do not know
Internal Partition	The presence of an internal partition between animals within the transport vehicle	Yes, no, don’t know
Height of partition	The design and extent of the partition	Full height, partial flush with floor, partial gap above floor, other/don’t know, no partition
Height at which equine tied	The height of the ring (or similar) to which the animal was tied	Eye level or above, between withers and eye level, between chest and withers, below chest, not tied, don’t know
CCTV fitted	Whether or not the compartment containing the animals was fitted with CCTV that could be observed by the driver/passenger	Yes, no, don’t know
*Journey details*		
Type of equines	Type of equines being transported at the time of the incident	Horse Pony Horse and donkey Horse and pony
Number of animals being transported		One, two, >two
Reason for travel	The reason for the journey in which the incident occurred	Leisure, competition/training, maintenance (health, yard, breeding), other/don’t know
*Incident details*		
Date of incident		Not specified, in the last 5 years, >5 years ago
Duration of travel before incident	How long the equine had been travelling prior to the incident	≤1 h, >1 h ≤ 4 h, >4 h, unspecified
Motion of vehicle when incident occurred	Whether or not the vehicle was moving	Stationary, moving, braking, unspecified
Type of incident	Main attributed cause signifying type of incident	Road traffic collision (RTC), horse behaviour issue (HB), transport vehicle malfunction (TVM), other/multiple (OM)
Equine behaviour before incident	If known, whether the equine was standing or moving	Standing still, fidgeting (inc. kicking, pawing, vocalising), unspecified
Incident considered avoidable	Whether measures could have been taken to avoid the occurrence	Yes, no
*Incident outcomes*		
Equine injured		Yes, no
Area of injury	Area on equine’s body that injury sustained	Multiple, head/neck, shoulder/torso/back, front legs, hind legs
Severity of injury		Minor, severe, fatal
Made full recovery	Recovered to pre-incident status	Yes, no
Time to full recovery		Not recovered, within 24 h, >24 h ≤ 1 week, >1 week ≤ 6 months, >6 months

**Table 3:** Frequency table for demographic and transport characteristics of survey population (count and percentage).

Survey Variable	Category	Count	Percentage
*Respondent details*			
Age	<26 years 26–55 years >55 years Missing data	235 1477 390 14	11.1 69.8 18.4 0.7
Gender	Male Female Missing data	118 1982 16	5.5 93.7 0.8
Involvement with equine industry	Recreation (R) Professional/competitive) (P) Multiple (M)	891 313 912	42.1 14.8 43.1
*Driver and vehicle details*			
Driver identity	Self Commercial Multiple drivers	1792 64 260	84.7 3.0 12.3
Driver training	Yes No	319 1797	15.1 84.9
Driver qualifications	Yes No	704 1412	33.3 66.7
Transport vehicle	Trailer Motorised horsebox Both Commercial transport only	1044 744 264 64	49.3 35.2 12.5 3.0
*Journey details*			
Reason for transport	Leisure and recreation (L) Competition and training (C) Multiple	130 96 1890	6.1 4.5 89.3
Number of animals transported	One Two >Two	1166 821 129	55.1 38.8 6.1
Frequency of journey	Weekly or more Every 2–4 weeks Less than once a month Varies	770 673 175 498	36.4 31.8 8.3 23.5
Duration of journey	<1 h 1–4 h >4 h	1220 841 55	57.7 39.7 2.6

**Table 4:** The counts and percentage breakdown of incidents and near misses (including the frequency of different types of near miss) reported by the survey population (n = 2116).

Survey Variable	Category	Count	Percentage
Incident	Yes No	342 1774	16.2 83.8
Number of incidents reported	None One Two Three Four Five	1774 290 48 3 1 0	83.8 13.7 2.3 0.1 <0.05 0
Near miss	Yes No	571 1545	27 73
Type of near miss	Other road users Horse/vehicle feature issues Vehicle malfunction Horse behaviour not related to vehicle features Horse falls Tying related issue Hay-net issue Weather Driver error	293 88 81 41 20 13 6 18 11	13.8 4.2 3.8 1.9 0.9 0.6 0.3 0.9 0.5
Near miss OR incident reported	Yes No	767 1349	36.2 63.8
Near miss AND incident reported	Yes No	146 1970	6.9 93.1

**Table 5:** Results of univariate logistic regression analyses of the significant association between the variable ‘involvement with equine industry’ and whether a near miss was reported.

Variable and Categories	Near Miss NO (n ^1^, %)	Near Miss YES (n ^1^, %)	OR ^2^	95% CI ^3^		*p* ^4^
				Lower	Upper	
*Variable: Involvement with equine industry*						
Recreation (R) Professional/competitive) (P) Multiple (M)	693 (32.75%) 224 (10.59%) 628 (29.68%)	198 (9.36%) 89 (4.21%) 284 (13.42%)	Ref 1.39 1.58	1.04 1.28	1.86 1.96	<0.001

^1^ Number of responses out of 2116 total participants; ^2^ Odds ratios (the odds of a participant reporting a near miss for each category compared with the reference category); ^3^ Confidence intervals; ^4^ Wald test *p*-value.

**Table 6:** Results of univariate logistic regression analyses of significant associations between variables (involvement with equine industry, main driver identity, reason for transport and frequency of journeys) and whether an incident was reported.

Variable and Categories	Incident NO (n ^1^, %)	Incident YES (n ^1^, %)	OR ^2^	95% CI ^3^		*p* ^4^
				Lower	Upper	
*Variable: Involvement with equine industry*						
Recreation (R) Professional/competitive) (P) Multiple (M)	782 (36.96%) 253 (11.96%) 739 (34.92%)	109 (5.15%) 60 (2.84%) 173 (8.18%)	Ref 1.70 1.68	1.20 1.30	2.40 2.18	<0.001
*Variable: Driver identity*						
Self Commercial Multiple drivers	1513 (71.5%) 58 (2.74%) 203 (9.59%)	279 (13.19%) 6 (0.28%) 57 (2.69%)	Ref 0.56 1.52	0.24 1.11	1.31 2.10	0.012
*Variable: Reason for transport*						
Leisure and recreation (L) Competition and training (C) Multiple	121 (5.72%) 85 (4.02%) 1568 (74.10%)	9 (0.43%) 11 (0.52%) 322 (15.22%)	Ref 1.74 2.76	0.69 1.39	4.38 5.49	0.006
*Variable: Frequency of journey*						
Less than once a month Every 2–4 weeks Weekly or more Varies	157 (7.42%) 579 (27.36%) 613 (29.00%) 425 (20.09%)	18 (0.85%) 94 (4.44%) 157 (7.42%) 73 (3.45%)	Ref 1.42 2.23 1.50	0.83 1.33 0.87	2.42 3.75 2.59	0.001

^1^ Number of responses out of 2116 total participants; ^2^ Odds ratios (the odds of a participant reporting an incident for each category compared with the reference category); ^3^ Confidence intervals; ^4^ Wald test *p*-value.

**Table 7:** Results of multivariate logistic regression analysis of associations between reporting an incident and the explanatory variables: involvement with the equine industry, driver identity and reason for transport.

Variable and Categories	Estimate	SE ^1^	OR ^2^	95% CI ^3^	*P* ^4^
*Variable: Involvement with the equine industry*					
Recreation (R) Professional/competitive) (P) Multiple (M)	Ref 0.45 0.40	0.18 0.14	Ref 1.57 1.50	1.11–2.23 1.15–1.95	0.006
*Variable: Driver identity*					
Self Commercial Multiple drivers	Ref -0.40 0.36	0.44 0.17	Ref 0.67 1.44	0.29–1.59 1.04–1.98	0.05
*Variable: Reason for transport*					
Leisure and recreation (L) Competition and training (C) Multiple	Ref 0.33 0.76	0.48 0.36	Ref 1.39 2.13	0.55–3.56 1.05–4.31	0.05

^1^ Standard error of the estimate; ^2^ Odds ratios (the odds of a participant reporting an incident for each category compared with the reference category); ^3^ Confidence intervals; ^4^ Wald test *p*-value.

**Table 8:** Frequency table for incident details (count and percentages for incident-specific driver and vehicle details, journey details and incident characteristics) for the 399 incidents reported.

Variable	Category	Count	Percentage
*Driver and vehicle details*			
Vehicle	Trailer Motorised horsebox	257 142	64.6 35.6
Vehicle owner	Own Commercial Borrowed/rented Friend	299 12 22 66	74.9 3.0 5.5 16.5
Driver held qualifications	Yes No Don’t know	112 235 52	28.1 58.9 13.0
Horsebox size (Horseboxes only)	>3.5 tonnes ≤3.5 tonnes Don’t know	92 44 6	23.1 11 1.6
Trailer servicing (Trailers only)	Within last 6 months 7–12 months before 1–5 years before Never serviced Don’t know	111 72 14 5 55	27.8 18 3.5 1.3 13.8
Internal Partition	Yes No Don’t know	375 21 3	94 5.3 0.8
Height of partition	Full height Partial height flush with floor Partial height with gap above floor Other/don’t know No partition	52 65 242 19 21	13 16.3 60.7 4.8 5.3
Height at which equine tied	Eye level or above Between withers and eye level Below withers Not tied Don’t know	237 104 33 12 13	59.4 26.1 8.3 3.0 3.3
CCTV fitted	Yes No Don’t know	78 313 8	19.5 78.4 2.0
*Journey details*			
Type of equines	Horse Pony Horse and donkey Horse and pony	285 87 1 26	71.4 21.8 0.3 6.5
Number of animals being transported	One Two >two	255 122 22	63.91 30.58 5.51
Reason for travel	Leisure Competition/training Maintenance Don’t know	64 219 75 41	16 54.9 18.8 10.3
*Incident details*			
Date of incident	In the last 5 years >5 years ago Missing data	177 130 92	44.4 32.6 23.1
Duration of travel before incident	≤1 h >1 h ≤ 4 h >4 h Missing data	261 46 5 86	65.4 11.5 1.3 21.6
Motion of vehicle when incident occurred	Stationary Moving Braking Missing data	15 269 9 106	3.8 67.4 2.3 26.6
Type of incident	Road traffic collision (RTC) Horse behaviour issue (HB) Transport vehicle malfunction (TVM) Other/multiple (OM)	36 222 23 118	9.0 55.6 5.8 29.6
Equine behaviour before incident	Standing still Fidgeting (kicking, pawing, vocalising) Missing data	200 170 29	50.1 42.6 7.3
Incident considered avoidable	Yes No Missing data	231 114 54	57.9 28.6 13.5

**Table 9:** The counts and percentage breakdown of incident outcomes (equine injury and recovery) in relation to the 399 incidents reported by survey respondents (n = 342).

Incident Outcomes			
Variable	Categories	Count	Percentage
Equine injured	Yes No	206 193	51.6 48.4
Area of injury	Multiple Head/neck Shoulder/torso/back Front legs Hind legs	83 23 20 18 82	20.9 5.8 5.0 4.5 20.6
Severity of injury	Minor Severe Fatal Missing data	126 68 8 4	31.6 17.0 2.0 1.0
Made full recovery	Yes No Missing data	170 35 1	42.6 8.8 0.3
Time to full recovery	Within 24 h >24 h ≤ 1 week >1 week ≤ 6 months >6 months Not recovered Missing data	3 35 81 10 30 57	0.8 8.8 20.3 2.5 7.5 14.25

**Table 10:** Results of the univariate logistic regression analyses of associations between incident characteristics (significant explanatory variables: type of equid, duration of journey at the time of the incident and the type of incident) and whether the incident resulted in equine injury.

Variable and Categories	Injury NO Count (%)	Injury YES Count (%)	OR ^1^	95% CI ^2^		*p* ^3^
				Low	High	
*Variable: Type of equid*						
Pony Horse	54 (14.52) 128 (34.41)	33 (8.87) 157 (42.21)	Ref 2.01	1.27	3.28	0.006
*Variable: Type of incident*						
Road traffic collision	28 (7)	8 (2)	Ref			0.002
Horse behaviour	96 (24.1)	126 (31.6)	4.59	2.01	10.52	
Transport vehicle malfunction	8 (2)	15 (3.8)	6.56	2.05	21.00	
Multiple	61 (15.3)	57 (14.3)	3.27	1.38	7.76	
*Variable: Duration of travel prior to incident*						
>one h ≤one h	34 (10.9)	18 (5.8)	Ref			0.023
	125 (39.9)	136 (43.5)	2.06	1.11	3.82	

^1^ Odds ratios (the odds of a participant reporting an injury for each category in comparison with the reference category); ^2^ Confidence intervals; ^3^ Wald test p-value.

**Table 11:** Results of multivariate logistic regression analysis of associations between injury and the explanatory variables: type of equid, type of incident, duration of travel prior to incident.

Variable and Categories	Estimate	SE ^1^	OR ^2^	95% CI ^3^	*p* ^4^
*Variable: Type of equid*					
Pony	Ref		Ref		0.003
Horse	0.86	0.30	2.37	1.33–4.23	
*Variable: Type of incident*					
Road traffic collision	Ref		Ref		0.007
Horse behaviour	1.35	0.45	3.84	1.61–9.19	
Transport vehicle malfunction	1.76	0.64	5.78	1.66–20.21	
Multiple	0.87	0.47	2.39	0.96–5.97	
*Variable: Duration of travel prior to incident*					
>1 h	Ref		Ref		0.096
≤1 h	0.56	0.34	1.75	0.91–3.40	

^1^ Standard error of the estimate; ^2^ Odds ratios (the odds of a participant reporting an injury for each category compared with the reference category); ^3^ Confidence intervals; ^4^ Wald test *p*-value.

## Supplementary Materials

The following are available online at https://www.mdpi.com/2076-2615/10/2/288/s1, S1: Survey questionnaire, Table S1: Attributed causes of incident and frequency of reported details of the specific type of occurrence. Table S2: Regional location of incident occurrence in England, Scotland and Wales for those incidents for which UK location details had been provided (n = 234).
